# Integration of Nanofiltration and Reverse Osmosis Technologies in Polyphenols Recovery Schemes from Winery and Olive Mill Wastes by Aqueous-Based Processing

**DOI:** 10.3390/membranes12030339

**Published:** 2022-03-18

**Authors:** Paulina Tapia-Quirós, María Fernanda Montenegro-Landívar, Mònica Reig, Xanel Vecino, Javier Saurina, Mercè Granados, José Luis Cortina

**Affiliations:** 1 Chemical Engineering Department, Escola d’Enginyeria de Barcelona Est (EEBE), Campus Diagonal-Besòs, Universitat Politècnica de Catalunya (UPC)-BarcelonaTECH, C/Eduard Maristany 10–14, 08930 Barcelona, Spain; paulina.tapia@upc.edu (P.T.-Q.); maria.fernanda.montenegro@upc.edu (M.F.M.-L.); monica.reig@upc.edu (M.R.); xanel.vecino@upc.edu (X.V.); 2 Barcelona Research Center for Multiscale Science and Engineering, Campus Diagonal-Besòs, 08930 Barcelona, Spain; 3 Centro de Investigación en Tecnologías, Energía y Procesos Industriales (CINTECX), Chemical Engineering Department, Campus As Lagoas-Marcosende, University of Vigo, 36310 Vigo, Spain; 4 Department of Chemical Engineering and Analytical Chemistry, Universitat de Barcelona, Diagonal 645, 08028 Barcelona, Spain; xavi.saurina@ub.edu (J.S.); mgranados@ub.edu (M.G.); 5 Water Technology Centre (CETAQUA), Carretera d’Esplugues 75, 08940 Cornellà de Llobregat, Spain

**Keywords:** resource recovery, phenolic compounds, nanofiltration, reverse osmosis, circular economy

## Abstract

More sustainable waste management in the winery and olive oil industries has become a major challenge. Therefore, waste valorization to obtain value-added products (e.g., polyphenols) is an efficient alternative that contributes to circular approaches and sustainable environmental protection. In this work, an integration scheme was purposed based on sustainable extraction and membrane separation processes, such as nanofiltration (NF) and reverse osmosis (RO), for the recovery of polyphenols from winery and olive mill wastes. Membrane processes were evaluated in a closed-loop system and with a flat-sheet membrane configuration (NF270, NF90, and Duracid as NF membranes, and BW30LE as RO membrane). The separation and concentration efficiency were evaluated in terms of the total polyphenol content (TPC), and by polyphenol families (hydroxybenzoic acids, hydroxycinnamic acids, and flavonoids), using high-performance liquid chromatography. The water trans-membrane flux was dependent on the trans-membrane pressure for the NF and RO processes. NF90 membrane rejected around 91% of TPC for the lees filters extracts while NF270 membrane rejected about 99% of TPC for the olive pomace extracts. Otherwise, RO membranes rejected more than 99.9% of TPC for both types of agri-food wastes. Hence, NF and RO techniques could be used to obtain polyphenol-rich streams, and clean water for reuse purposes.

## 1. Introduction

Due to the current environmental situation, increasingly more agro-industries are dedicating their time and resources to minimize the waste generated, not only by optimizing their production processes but also by revaluing and reusing this waste at the end of the process, contributing to the concept of a circular economy [1]. Agri-food wastes are phytotoxic and a major environmental concern; so, their disposal is an important problem for industries, due to the environmental impact and high disposal costs [2,3].

Wine production involves the processing of more than 60 million tons of grapes per year [2,4]. The processing stages generate up to 30%, *w/w* of the raw material [4]. Among the different types of waste generated, wine lees are removed from the bottom of the wine containers after fermentation and along the aging stage [4]. Wine lees, mainly composed of bacteria, yeasts, tartaric acid, and polyphenols, could represent up to 3–7% (*v*/*v*) of wine production [4,5]. On the other hand, olive oil is one of the preferred edible oils for daily food uses. It is mainly produced in Europe, with 98% of the world production of 2.55 million metric tons per year [6]. The main solid waste generated is recognized as olive pomace. Olive pomace is only produced with traditional three and two-phase olive oil extraction systems [7]. These wastes are composed of olive pulp, stone, skin, oil, and water, with protein, polysaccharides, pigments, fatty acids, and polyphenols being the main compounds [8,9]. As wine and olive oil production are two important high-volume waste industrial sectors in southern Europe, both have been identified to develop and promote bio-based economy solutions [10]. Their processing residues are toxic to the environment but at the same time are characterized by their high-content bioactive molecules, such as polyphenols [4,7].

Polyphenolic compounds are considered secondary metabolites of vegetal species, which are chemically characterized by the presence of more than one phenolic group per molecule. They have been recognized by their properties of reducing and/or preventing the generation of free radicals involved in most of the human body’s oxidation processes [11]. Their strong antioxidant capacity and the potential associated health benefits could be applied in the food and beverage, cosmetic, and pharmaceutical industries [5]. Polyphenols can be classified into four large groups: phenolic acids (hydroxybenzoic and hydroxycinnamic acids), flavonoids (flavones, flavonols, flavanones, isoflavones, anthocyanidins) and flavanols (e.g., proanthocyanidins and catechins), lignans, and stilbenes [12]. As an example, syringic acid is a phenolic compound that belongs to the family of hydroxybenzoic acids. It is commonly found in winery by-products [4,13,14,15,16,17,18,19,20], and it is an antioxidant with neuroprotective, anti-glycating, anti-angiogenic, anti-hyperglycemic, and memory-enhancing properties with potential utility in pharmaceutical applications [21]. Moreover, 3-hydroxytyrosol is a phenolic compound that belongs to the group of phenyl alcohols and it is one of the major components in olive mill residues [9,22]. Its properties, such as antioxidant, antimicrobial, health-promoting, and disease-preventing abilities, have already been demonstrated [6,9,22,23,24,25]. In the food industry, 3-hydroxytyrosol showed favorable results as an antioxidant agent for active food packaging [26]. Moreover, 3-hydroxytyrosol recovered from olive mill residues has the potential to be used as a UV filter in cosmetic applications [23,27].

The possibility of introducing and commercializing such a list of polyphenols is associated with quality concerns in terms of purity. If polyphenols are to be used in human food applications and cosmetic applications, the purity values should be above 95% as then their presence in formulations is associated with claims fulfilling the food and drug regulations [28,29,30]. When polyphenols have lower purities and are also mixtures of different polyphenols, their applications are directed to animal feed and consequently the value of such polyphenols is decreased dramatically [31]. For this reason, the main challenge within the recovery and valorization of polyphenols is the need to achieve such high-purity by-products by developing requested processing trains from the original solid and liquid wastes.

Extraction is the first step to separate target compounds (e.g., polyphenols) from a solid matrix [32]. Solid–liquid extraction (SLE) is a widely applied extraction method used for thermolabile compounds, such as polyphenols, and it is based on the use of solvents to dissolve solutes from the matrix and transfer them to the solution. Widely used solvents include water, ethanol, methanol, ethyl acetate, and mixtures of them [33,34,35,36,37]. Nevertheless, water and ethanol are the most compatible options for the food, cosmetic, and pharmaceutical fields [10,32,38].

After the SLE process, a separation step is required to recover the extracted polyphenols from the complex solutions generated since other plant metabolites are also present. For this, membrane separation processes can be used at a large scale, with three main objectives: (i) to remove suspended solids and colloids from the SLE stage by using pressure-driven membrane techniques, such as microfiltration (MF) and ultrafiltration (UF); (ii) to selectively separate polyphenols from other families of compounds co-extracted in the SLE (e.g., sugars, proteins, lipids) using ultrafiltration (UF) and nanofiltration (NF); and iii) to concentrate the polyphenol-rich streams using nanofiltration (NF) and reverse osmosis (RO) [8,38,39,40,41,42,43,44,45]. Membranes are able to introduce separation factors according to their molecular weight cut-off (MWCO) (e.g., UF) or according to the solute and active layer properties (e.g., NF and RO) [42]. For both types of membranes, solute transport mechanisms can be described by the solution-diffusion model, where the solute distribution stage from the aqueous phase to the membrane phase is one of the relevant stages describing the permeation and/or rejection ratios. In particular, NF is a primary technology postulated for the separation and concentration of polyphenols [46]. NF membranes are dense membrane with free volume sizes ranging from 0.06 to 0.15 nm, rejecting solutes with MWCO from 100 to 1000 Da; therefore, they can separate and concentrate low-molecular-weight polyphenols [43,46]. However, when a volume reduction in the polyphenol-rich stream is pursued, the application of RO treatment is also necessary [46]. RO membranes are characterized by pore sizes less than 0.07 nm and MWCO of 1–100 Da, and they are used for concentration and stream reduction purposes [45]. Therefore, NF along with RO treatments can obtain concentrated polyphenol streams and high-quality water for reuse purposes (e.g., for the SLE stage). For instance, Zagklis et al. 38] evaluated a purification process for the recovery of polyphenols from grape marc, olive mill wastewater, and olive leaves residues. The proposed process was based on the following stages: (i) a solid-liquid extraction for solid by-products (50% *v*/*v* ethanol/water for grape marc; and water for olive leaves); (ii) a membrane separation treatment train including a tubular ceramic UF membrane, followed by polymeric NF and RO membranes in a spiral wound configuration; and (iii) a final adsorption step using polymeric adsorbents. As a result, 378, 190, and 98 g L^−1^ of phenols in gallic acid equivalents (GAE) were obtained for olive mill wastewater, grape marc, and olive leaf extracts, respectively. Giacobbo et al. [47] evaluated a sequential design of UF (ETNA01PP and ETNA10PP membranes) and NF (NF270 membrane) to fractionate polyphenols and polysaccharides from wine lees. UF was effective at separating the polysaccharide fraction from the polyphenol fraction while the subsequent NF membrane stage rejected more than 92% of the polyphenols, resulting in solutions with a high antioxidant capacity. Bottino et al. [41] integrated an MF stage (Membralox EP19–40 membrane) followed by an RO stage (SW30HR membrane) in the treatment of olive mill wastes. As a result, a polyphenol recovery ratio ˃ 99.3% was obtained. The permeate stream quality was adequate for on-site re-use.

The integration of a more sustainable extraction stage using aqueous solutions followed by a membrane separation and/or concentration stage has scarcely been studied. Cassano et al. [39] studied the extraction of polyphenols from wine lees using aqueous solutions (25% *w/w* during 60 min at 45 °C) along with a sequential membrane treatment train including: (i) polyvinylidenefluoride (PVDF) hollow-fiber MF-membranes and (ii) NP010, NP030, and MPF36 NF membranes. Nunes et al. [8] proposed an integrated scheme for polyphenol extraction from olive pomace wastes using water as the solvent (1:40 *w*/*v* for 2 h at 40 °C and 600 rpm) followed by the membrane treatment stage, incorporating 3 types of polymeric thin-film composite (TFC) membranes (NF90, NF270, and BW30). Moreover, Conidi et al. [48] investigated the integration of a combination of an aqueous extraction stage (1:5 *w*/*v* for 60 min at 70 °C) followed by a filtration stage incorporating the following separation processes for the recovery of polyphenols from olive mill solid wastes: (i) MF (MD 020 TP 2N membrane), (ii) UF (GK, GH, GE membranes), and iii) NF (NFA-12A, DK membranes).

On the other hand, there are other technologies, such as membrane distillation crystallization, that achieve the recovery of water and valuable solids. Membrane distillation is a thermally driven separation process, which involves a phase change where the thermal gradient between the feed and permeate is the driving force [49,50]. This process along with crystallization produces clean water and precipitates/crystallizes solids [50]. However, these separation processes could cause degradation of the compounds of interest (e.g., polyphenols) and high temperatures should thus be avoided. Therefore, the advantages of pressure-driven membrane technologies include low-temperature operation and low energy consumption, moderate separation factors, simple equipment, and easy scale-up [11].

In view of the foregoing, the current study evaluated a green extraction and purification process for polyphenol recovery from winery and olive mill industrial residues, based on: i) a solid-liquid extraction with water and ii) the use of nanofiltration and reverse osmosis technologies to achieve the separation of unwanted families of plant metabolites to increase the polyphenols content and reduce the streams for treatment in subsequent stages. It should be highlighted that the integration of NF processes in the processing of agro-food wastes is an approach that is under development and scarce studies could be found in the literature, although laboratory studies, such as this work, are being reported. This work presents some novel issues: i) the flat-sheet membrane configuration used has a bigger membrane area than the ones already tested in published papers; ii) an integration scheme, based on water extraction and membrane filtration processes (NF and RO), has not previously been tested for lees filters in a flat-sheet configuration; and iii) the olive pomace aqueous extraction conditions used and membrane type were different from that shown in the literature.

## 2. Materials and Methods

### 2.1. Reagents

The following polyphenols standards were used in this study: gallic acid, rutin, vanillic acid, 3,4-dihydroxybenzoic acid, syringic acid, chlorogenic acid, 3,4-dihydroxybenzaldeyde, ethyl gallate, 4-hydroxybenzoic acid, ferulic acid, epicatechin, naringenin, *p*-coumaric acid, 2,5-dihydroxybenzoic acid, quercetin, and apigenin, and were supplied by Sigma Aldrich (St. Louis, MO, USA). Caffeic and caftaric acids were purchased from Chengdu Biopurify Pytochemicals (Chengdu, China). Catechin, 3-hydroxytyrosol, myricetin, and resveratrol were obtained from TCI (Tokyo, Japan). Oleuropein and homogentisic acid were purchased from Extrasynthese (Genay, France). Luteolin was obtained from Carbosynth (Berkshire, UK). Hesperidin and kaempferol were supplied by Glentham Life Sciences (Corsham, UK). The solvents used were HPLC-UV-grade acetonitrile (ACN) from Fisher Scientific (Madrid, Spain), HPLC-UV-grade ethanol (EtOH) obtained from Honeywell Riedel-de HaënTM (Seelze, Germany), and formic acid (FA) 98–100% (*w*/*w*) from Merck (Darmstradt, Germany). Ultrapure water was produced by a Milli-Q system (Merck Millipore).

### 2.2. Samples

Samples of lees filters and olive pomace residues were collected from different relevant Spanish winery and olive oil producer companies. Lees filters were obtained from red wine production using a combination of Tempranillo, Cariñena, Garnacha, and Cabernet Sauvignon grapes. Samples were collected in the period between August and October 2018, in a winery from Barcelona, Spain. Olive pomace samples were acquired from processing mills treating Hojiblanca and Picual olives and using a two-phase extraction system. Sampling was carried out throughout October 2018 and January 2019, in an olive oil industry from Córdoba, Spain. Both winery and olive mill residues were kept at −20 °C before use to preserve their properties.

### 2.3. Procedures

#### 2.3.1. Polyphenol Extraction

Polyphenols were extracted from lees filters and olive pomace based on a previous optimized solid-liquid extraction (SLE) methodology [51]. Ultrapure water was used as a means of extraction at a stirring rate of 300 rpm in an RCT basic hot plate stirrer with a temperature controller (IKA, Staufen, Germany). The extraction conditions for the lees filters were as follows: 70 °C for 10 min and a 1:100 (*w*/*v*) solid-to-liquid ratio. For olive pomace, the extraction conditions were: 25 °C for 10 min and a 1:30 (*w*/*v*) solid-to-liquid ratio. The extracts were centrifuged for 15 min at 3500× *g* rpm in a Labofuge 400 centrifuge (Heraeus, Hanau, Germany). Afterwards, the supernatants were filtered through 0.45 μm nylon syringe filters (Filtros Anoia, Barcelona, Spain) and stored at 4 °C before the analysis.

#### 2.3.2. Membrane Experimental Set-Up and Operation

**Figure 1:**
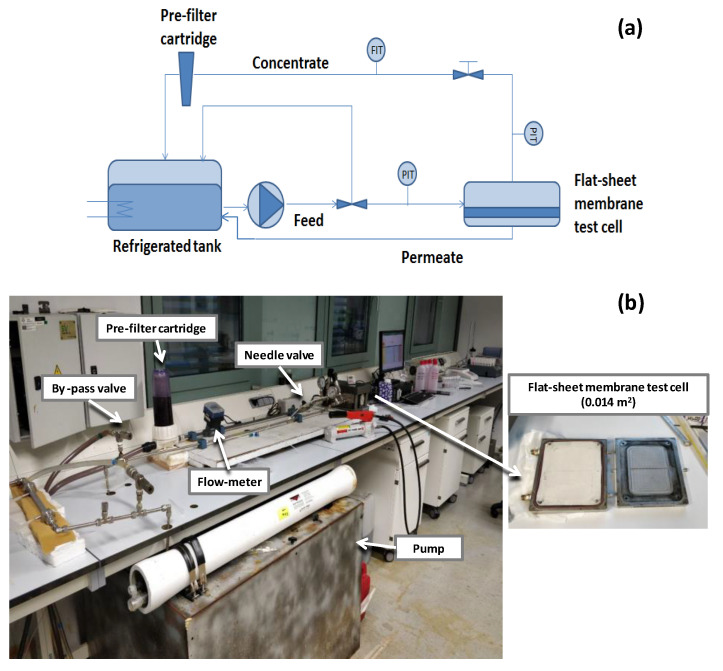
(**a**) NF and RO membrane filtration experimental set-up scheme and (**b**) a picture of the experimental set-up, for the evaluation of polyphenol rejection.

The membrane separation experiments were carried out in a crossflow configuration using a closed-loop mode where the concentrate and permeate streams are recirculated into the feed tank to maintain a feed solution with a constant concentration. For this, an SEPA™ CF II crossflow cell in the flat-sheet configuration mode (GE, PA, USA), with a spacer-filled feed channel, an active area of 0.014 m^2^, and 0.864 mm of gap thickness, equipped with a high-pressure diaphragm pump (Hydra-Cell, MN, USA) was used. Details of the experimental set-up (scheme and picture) are described in Figure 1.

Table 1 shows the studied membranes and their characteristics. The membranes tested for the NF and RO processes were TFC dense membranes. For the NF technique, the membranes evaluated were polypiperazine thin-film composite (NF270), polyamide (NF90), and a sulfonamide active layer and polysulfone support (Duracid). Regarding the RO technique, the membrane evaluated was a polyamide thin-film composite (BW30LE).

NF and RO membranes were soaked in deionized Milli-Q water overnight before experiments to remove conservative products and impurities, and to densify the membrane support layer. Afterwards, the membranes were pressurized with de-ionized water at 22 bar (TMP), and 5 mL min^−1^ of feed flow, for 2 h. The same procedure was repeated with the feed solution to ensure that the membrane density was constant during the whole experiment. In other words, pressurization was performed prior to the experiment to ensure the densification of the active layer of the membrane and to minimize irreversible membrane compaction during the ion rejection measurements. A 5-L feed solution refrigerated by a water bath (˂30 °C) was pumped into the crossflow cell at a cross-flow velocity of 1 m s^−1^. The system had a by-pass valve and a needle to vary the trans-membrane pressure (TMP). A pre-filter cartridge was placed to protect the pump and the membrane from fouling by erosion products. A sample of the initial solution was taken, and the experiment was carried out by varying the TMP from the osmotic pressure to 20 bar (in 7 points) at a feed flow of 3.46 mL min^−1^. At each TMP increment, a permeate sample was collected by a three-way valve to measure temperature, pH (GLP22 pH-meter CRISON (Barcelona, Spain)), conductivity (GLP31 conductivity-meter CRISON (Barcelona, Spain)), and polyphenol concentrations by HPLC-UV. Moreover, at the end of the experiment a feed solution sample was taken to corroborate its constant composition during the experiment. Finally, a short cleaning step with water for 30 min at 10 bar and 5 mL min^−1^ was performed, followed by a long cleaning step with water for 1.5 h at 22 bar and 5 mL min^−1^. The assays were performed in duplicate for each membrane tested.

#### 2.3.3. Polyphenol Analysis by HPLC with UV Detection (HPLC-UV)

The polyphenolic content analysis was performed in an Agilent Series 1200 system equipped with a diode array detector, an automatic injection system, a quaternary pump, and an AgilentChemStation software (CA, USA). The chromatographic separation was carried out with a Kinetex C_18_ column (Phenomenex, 100 mm × 4.6 mm × 2.6 μm, Torrance, CA, USA), with ultrapure water with 0.1% FA (A) and ACN (B) as the mobile phase components, and a gradient profile described previously [10]. The injection volume and flow rate were 5 μL and 0.4 mL min^−1^, respectively. Chromatograms were recorded at 280, 310, 370, and 550 nm. The total polyphenol content (TPC) was determined from the chromatographic peaks area at 280 nm, in the time range between 5 and 36 min. The hydroxybenzoic acid (HB) content was estimated at 280 nm, in the time framework between 5 and 15 min for lees filters extracts, and from 7 to 14 min for olive pomace extracts; hydroxycinnamic acid (HC) content at 310 nm, in the time range between 15 and 21 min for lees filters extracts, and from 14 to 23 min for olive pomace extracts; and flavonoids (F) content at 370 nm in the time range between 21 and 36 min for lees filters extracts, and from 23 to 42 min for olive pomace extracts. TPC and HB were expressed in terms of mg L^−1^ of gallic acid equivalent (mg GAE L^−1^), HC was expressed in terms of mg L^−1^ of caffeic acid equivalent (mg CAE L^−1^), and F was expressed in terms of mg L^−1^ of kaempferol equivalent (mg KE L^−1^). Calibration curves were constructed at concentrations from 0.5 to 10 mg L^−1^ for gallic acid, caffeic acid, and kaempferol.

### 2.4. Membrane Separation Operational Parameters

The membranes’ effectiveness was determined by different parameters. The TMP was calculated by Equation (1) [56]:

(1) $$\mathrm{TMP}\;\left(\mathrm{bar}\right)=\frac{P_{in}+P_{out}}{2}-P_{p}$$

where *P_in_* is the feed stream pressure (bar), *P_out_* is the concentrate stream pressure (bar), and *P_p_* is the permeate stream pressure (bar).

The trans-membrane flux (*Jv*) was also calculated by Equation (2) [56]:

(2) $$Jv\left(\frac{L}{m^{2}\cdot h}\right)=\frac{Q_{p}}{A_{m}}$$

where *Q_p_* is the permeate flow (L h^−1^) and *A_m_* is the active filtration area (0.014 m^2^).

Moreover, the rejection (*R*) percentage was estimated by Equation (3) [56]:

(3) $$R\left(\%\right)=\frac{C_{f}-C_{p}}{C_{f}}\cdot 100$$

where *C_f_* is the feed polyphenol concentration (mg L^−1^) and *C_p_* is the permeate polyphenol concentration (mg L^−1^).

### 2.5. Data Analysis

One-factor analysis of variance (ANOVA) with replication at the 95% confidence level (*p* < 0.05) was applied to statistically evaluate the differences between the tested membranes. Microsoft Excel 2019 was used for ANOVA analysis.

## 3. Results and Discussion

### 3.1. Polyphenol Extracts from Agri-Food Residues

The lees filters extract’s initial composition was 33 mg L^−1^ of TPC, with a polyphenol composition of 6.8, 4.5, and 4.6 mg L^−1^ of HB, HC, and F, respectively. Table 2 summarizes the main polyphenols identified: syringic acid (4.3 ± 0.1 mg L^−1^), hesperidin (3.0 ± 0.2 mg L^−1^), gallic acid (1.7 ± 0.1 mg L^−1^), 3,4-dihydroxybenzoic acid (0.6 ± 0.1 mg L^−1^), and *p*-coumaric acid (0.5 ± 0.1 mg L^−1^). Regarding the olive pomace extracts, TPC’s initial composition was 173 mg L^−1^; and HB, HC, and F was 23.1, 8.2, and 37.1 mg L^−1^, respectively. The main polyphenols identified were oleuropein (17.1 ± 0.7 mg L^−1^), 3-hydroxytyrosol (4.1 ± 0.1 mg L^−1^), 4-hydroxybenzoic acid (3.6 ± 0.1 mg L^−1^), *p*-coumaric acid (1.9 ± 0.1 mg L^−1^), and rutin (1.8 ± 0.1 mg L^−1^) (Table 2).

### 3.2. Treatment of Aqueous Extracts with NF Membranes

The nanofiltration membranes NF90 and Duracid were selected for the lees filters extracts and NF270 membrane for the olive pomace extracts, based on previous works (data not shown), to evaluate the polyphenols’ separation [51].

#### 3.2.1. NF Trans-Membrane Flux Analysis

**Figure 2:**
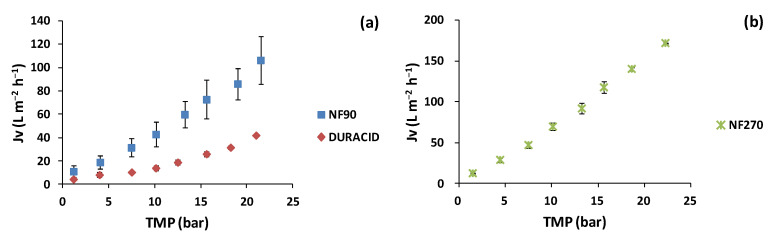
Variation in the trans-membrane flux as a function of TMP for (**a**) lees filters with NF90 and Duracid membranes and (**b**) olive pomace extracts with NF270 membrane.

The variation in the trans-membrane flux (*Jv*) as a function of TMP was studied for the NF membranes. Figure 2 shows a linear relationship where, as TMP increased, *Jv* also increased. This relationship varied according to the nature of the tested membrane and the evaluated matrix.

Figure 2a shows the results for the lees filters extracts. For the NF90 membrane, *Jv* increased from 11.1 ± 4.9 to 106.5 ± 20.1 L m^−2^ h^−1^ in the TMP range of 1 to 22 bar. On the other hand, for the Duracid membrane, *Jv* increased from 4.4 ± 1.1 to 42.0 ± 0.4 L m^−2^ h^−1^ in the same TMP range. It can be observed that the lees filters extract showed higher *Jv* values with the NF90 membrane compared to with the Duracid membrane in the tested TMP range. Therefore, for the Duracid membrane, a significant increase in TMP is needed for major trans-membrane flux. For the olive pomace extracts (Figure 2b), the NF270 membrane also showed a lineal increase in *Jv* from 12.5 ± 0.3 to 171.9 ± 0.6 L m^−2^ h^−1^ in the TMP range of 1 to 22 bar.

The NF270 membrane showed higher *Jv* values for olive pomace extracts when compared with the NF90 and Duracid membranes for the lees filters extracts. NF270 had the highest MWCO (400 Da), followed by NF90 (MWCO 200 Da), and Duracid (MWCO 150–200) [56]. Nunes et al. [8] compared the NF270 and NF90 membranes with olive pomace aqueous extracts with an effective filtration area of 52 cm^2^ and obtained higher permeate fluxes with the NF270 membrane due to the lower hydraulic resistance to water transport by the relatively loose semi-aromatic active layer. Similarly, Giacobbo et al. [57] obtained a linear increase in the permeation fluxes with the TMP in winery wastewaters using an NF270 membrane of 300 Da MWCO.

#### 3.2.2. NF Polyphenol Rejection Analysis

To test the polyphenol separation capacity of the tested membranes, a rejection analysis of TPC and by the polyphenol families (hydroxybenzoic acids (HB), hydroxycinnamic acids (HC), and flavonoids (F)) was carried out. The TPC and the three polyphenols’ content were analyzed according to Section 2.3.3.

**Figure 3:**
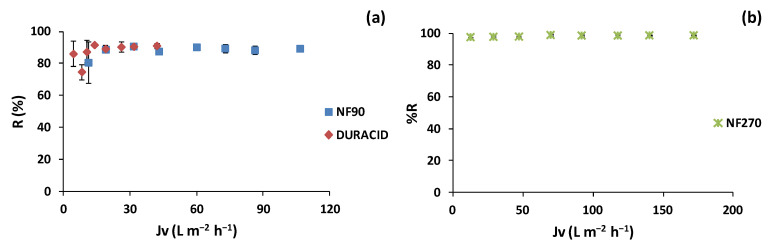
Polyphenol rejection evolution with trans-membrane flux for (**a**) lees filters with NF90 and Duracid membranes and (**b**) olive pomace extracts with NF270 membrane.

The results of the TPC rejection for the lees filters extracts are shown in Figure 3a. It can be seen that for both membranes, as *Jv* increased, the rejection percentage increased, reaching a maximum rejection point where it remained stable. The NF90 membrane obtained a maximum TPC rejection of 90.8 ± 0.4% at *Jv* of 31.5 L m^−2^ h^−1^ (7 bar of TMP). The rejection percentage remained stable at *Jv* of 19.0 L m^−2^ h^−1^ (4 bar TMP) at ca. 90% of rejection up to the maximum *Jv* of 106.5 L m^−2^ h^−1^ (22 bar of TMP). The TPC average rejection in the studied range was of 88.1 ± 4.0%. On the other hand, the highest TPC rejection obtained by the Duracid membrane was 91.7 ± 0.5% at*Jv* of 14.0 L m^−2^ h^−1^ (10 bar), remaining stable up to the maximum *Jv* of 42.0 L m^−2^ h^−1^ (22 bar). The average rejection obtained in the studied range was 87.4 ± 5.5%. The results are in concordance with the literature. Kontogiannopoulos et al. [5] obtained 98% polyphenol rejection using an NF90 membrane with an active filtration area of 12.6 cm^2^ for wine lees diluted in water and acidified at pH 3. Moreover, Cassano et al. [58] obtained polyphenols rejection of 93% with an NF90 membrane with an effective membrane surface area of 38.5 cm^2^ for olive mill wastewater using a bench laboratory set-up equipped with a spiral-wound membrane module. ANOVA assessment showed that there was no significant difference between the 2 tested NF membranes regarding the recovery of polyphenols (*p* = 0.82). Nevertheless, the NF90 membrane required less TMP to obtain a higher trans-membrane flux than the Duracid membrane under the studied conditions.

For the olive pomace extracts (Figure 3b), the NF270 membrane reached the highest TPC rejection of 99.0 ± 0.6% at *Jv* of 69.6 L m^−2^ h^−1^ (10 bar of TMP) and remained stable up to the maximum *Jv* of 171.9 L m^−2^ h^−1^ (22 bar of TMP). This is in accordance with Nunes et al. [8], who obtained a TPC removal of 99.6% with an NF270 flat-sheet membrane for olive pomace aqueous extracts. Moreover, Ioannou-Ttofa et al. [59] achieved TPC removal up to 95% with an NF270 flat-sheet membrane with a filtration area of 120 cm^2^ for olive mill wastewater treatment.

**Figure 4:**
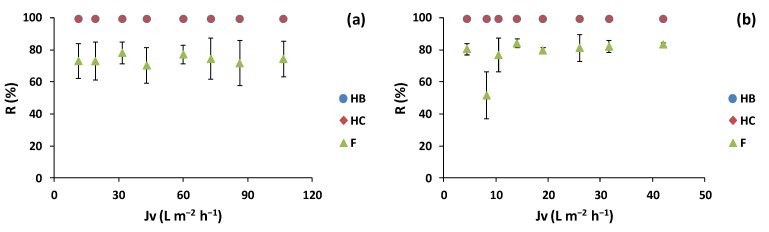
Polyphenol families’ rejection of hydroxybenzoic acids (HB), hydroxycinnamic acids (HC), and flavonoids (F) with trans-membrane flux for lees filters extracts with (**a**) NF90 and (**b**) Duracid membranes.

The results of the polyphenol families’ rejection for the lees filters extracts are shown in Figure 4.

The results indicated a similar behavior for the NF90 and Duracid membranes. Both membranes obtained concentrations of HB and HC families in the permeate streams below the HPLC-UV limit of quantification (LOQ < 0.5 mg L^−1^). Regarding the flavonoid family, the NF90 membrane obtained a maximum F rejection of 78.5 ± 6.9% at a trans-membrane flux of 31.5 L m^−2^ h^−1^ (7 bar) and an average rejection of 74.1 ± 2.6%. On the other hand, the Duracid membrane obtained a maximum F rejection of 84.2 ± 2.8% at*Jv* of 14.0 L m^−2^ h^−1^ (10 bar) and an average rejection of 81.2 ± 2.5%. In the literature, Cassano et al. [39] showed anthocyanin rejection higher than 93% with NF membranes (NP010, NP030, and MPF36 membranes) from red wine lees aqueous extracts.

**Figure 5:**
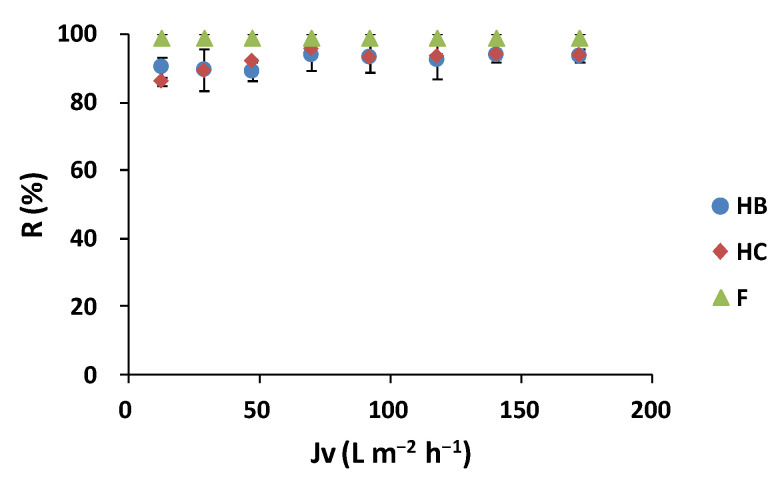
Polyphenol families (hydroxybenzoic acids (HB), hydroxycinnamic acids (HC) and flavonoids (F)) rejection with trans-membrane flux for olive pomace extract with NF270 membrane.

For the olive pomace extracts (Figure 5), simila to the TPC analysis, the highest rejection values for the HB and HC families were obtained at 69.6 L m^−2^ h^−1^ (10 bar TMP), with rejection values of 94.1 ± 4.7% for HB and 95.8 ± 2.1 for HC. On the other hand, the F rejection was 100% throughout the experiment, obtaining F concentrations in the permeate streams below the LOQ of the HPLC-UV technique (LOQ < 1 mg L^−1^). Similarly, Conidi et al. [48] rejected 100% of the flavanols and hydroxycinnamic acid derivatives with the NFA-12A and DK NF membranes at 25 bar in aqueous extracts of olive mill solid wastes.

### 3.3. Treatment of Aqueous Extracts with RO Membrane Experiments

The reverse osmosis BW30LE membrane was tested for the lees filters and olive pomace extracts as a final purification step.

#### 3.3.1. RO Trans-Membrane Flux Analysis

**Figure 6:**
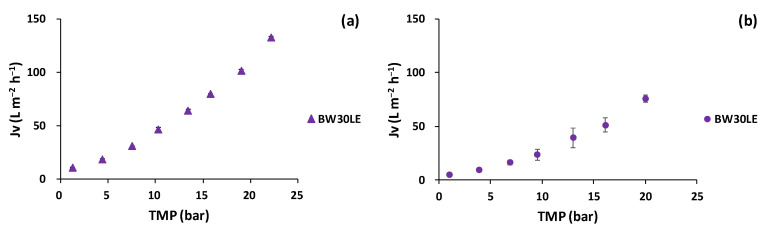
Trans-membrane flux variation as a function of the trans-membrane pressure for BW20LE RO membrane for (**a**) lees filters and (**b**) olive pomace extracts.

The results of the trans-membrane flux evolution as a function of TMP are shown in Figure 6.

The results showed that as TMP increasesd, the *Jv* values increased for both extracts. For the lees filters extract (Figure 6a), *Jv* increased from 10.8 ± 0.4 to 133.0 ± 1.3 L m^−2^ h^−1^ in the TMP range of 1 to 22 bar. For the olive pomace extract, a lower *Jv* was obtained from 5.1 ± 0.9 to 76.0 ± 3.0 L m^−2^ h^−1^ in the same TMP range (Figure 6b). The *Jv* differences between the lees filters and olive pomace extracts can be explained due to the different concentrations of extracts, as the olive pomace extracts were more concentrated (in terms of TPC) than the lees filters extracts. The ANOVA assessment indicated that there was no significant difference in the BW30LE membrane between the 2 studied extracts (*p* = 0.14). In the literature, Nunes et al. [8] acquired *Jv* values of ≈15 L m^−2^ h^−1^ at 20 bar for aqueous olive pomace extracts treated with a flat-sheet BW30LE membrane with an effective filtration area of 51.4 cm^2^.

#### 3.3.2. RO Polyphenols Rejection Analysis

**Figure 7:**
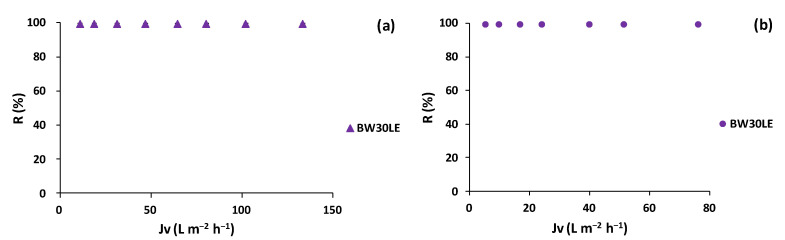
Polyphenol rejection variation as a function of the trans-membrane flux for BW30LE RO membrane for (**a**) lees filters and (**b**) olive pomace extracts.

The polyphenol rejection analysis with the BW30LE membrane was also tested for the lees filters and olive pomace extracts. As a result, for both extracts, the BW30LE membranes rejected 100% of the TPC-obtaining permeate streams with polyphenol concentrations below LOQ of the HPLC-UV method (Figure 7).

**Figure 8:**
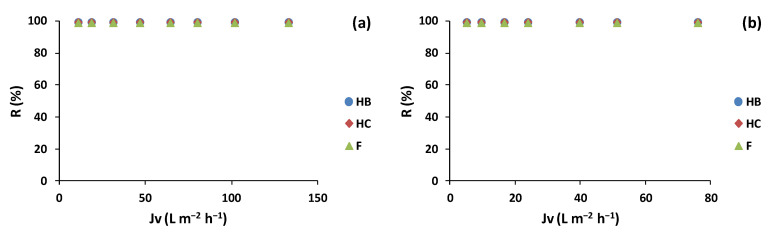
Polyphenol families (hydroxybenzoic acids (HB), hydroxycinnamic acids (HC), and flavonoids (F)) rejection with trans-membrane flux for (**a**) lees filters and (**b**) olive pomace extracts with BW30LE membrane.

Regarding the polyphenols families’ (HB, HC, and F) rejection (Figure 8), the same results were obtained for TPC for the lees filters and olive pomace extracts. The BW30LE membranes rejected 100% of the polyphenol families, obtaining permeate streams with polyphenols concentrations below LOQ of the HPLC-UV method. This behavior is in agreement with Nunes et al. [8], who obtained 100% rejection for phenolic compounds and flavonoids from aqueous olive pomace extracts filtered with BW30LE membranes. Moreover, Bottino et al. [41] obtained 99.2% phenolic compound rejection with a flat-sheet BW30LE membrane (surface 0.0066 m^2^) for olive mill wastewater at 30 bar and a feed flow rate of 1000 L h^−1^.

Concretely, as an example of the individual polyphenols’ rejection by membrane technology (NF and RO), 3-hydroxytyrosol was rejected by 86.9 ± 3.9% in the olive pomace extracts and syringic acid was fully rejected (100 ± 0.1%) in the lees filters extracts with the NF270 and BW30LE membranes, respectively. The recovery of these purified compounds by membrane technology is very attractive for the olive mill and winery industries to follow the circular economy framework and constitutes a key point in obtaining products of industrial interest (e.g., polyphenols), promoting an industrial synergy.

## 4. Conclusions

In general, high rejection factors of polyphenols using NF and RO processes in closed-loop systems were measured for winery and olive mill industrial residues, e.g., lees filters and olive pomace extracts, respectively. Three NF (NF270, NF90, and Duracid) and one RO (BW30LE) membranes were tested for trans-membrane flux and polyphenols recovery analysis. For the NF and RO processes, the trans-membrane flux was dependent in the TMP under the tested conditions.

For the lees filters extracts, the NF90 membrane rejected around 91% of TPC at a lower TMP (7 bar) and higher *Jv* (31.5 L m^−2^ h^−1^) than the Duracid membrane (92% of TPC rejection at 10 bar and 14.0 L m^−2^ h^−1^). Nevertheless, the Duracid membrane rejected more flavonoids (84%) than the NF90 membrane (79%). For the olive pomace extracts, the NF270 membrane rejected 99% of TPC at 10 bar of TMP and 69.6 L m^−2^ h^−1^ of *Jv*. Moreover, 100% of flavonoids were rejected under the same conditions.

On the other hand, RO membranes (BW30LE) rejected 100% of the polyphenols obtained as permeates, with a polyphenol content below LOQ of the HPLC-UV technique (LOQ = ˂ 0.5 mg L^−1^ for HB and HC, and ˂ 1 mg L^−1^ for F). The RO technique was able to obtain polyphenol-rich streams and clean water for reuse purposes. Consequently, the NF and RO processes applying NF90 and BW30LE as the nanofiltration and reverse osmosis membranes, respectively, for lees filters extracts and NF270 and BW30LE for the olive pomace extracts could be an attractive method for separating and concentrating polyphenols from winery and olive mill wastes using aqueous-based processing.

## Figures and Tables

**Table 1:** The membranes used in this study and their characteristics.

Membrane	Manufacturer	Membrane Composition	MWCO (Da)	pH Range (at 25 °C)	Max. Pressure (bar)	Max. Temperature (°C)
**NF**						
NF270 [52]	DuPont-Filmtec	Polypiperazine Thin-Film Composite	400	2–11	41	45
NF90 [53]	DuPont-Filmtec	Polyamide Thin-Film Composite	200	2–11	41	45
Duracid [54]	Suez	Sulfonamide active layer and polysulfone support	150–200	˂9	83	70
**RO**						
BW30LE [55]	DuPont-Filmtec	Polyamide Thin-Film Composite	100	3–11	41	45

**Table 2:** Composition of polyphenols in the lees filters and olive pomace extracts.

Agri-Food Residue	Type of Polyphenol	Polyphenol Concentration (mg L^−1^)	Polyphenol Molecular Weight (Da)
Lees filters	Syringic acid	4.3 ± 0.1	198.2
	Hesperidin	3.0 ± 0.2	610.2
	Gallic acid	1.7 ± 0.1	170.1
	3,4-dihydroxybenzoic acid	0.6 ± 0.1	154.1
	*p*-coumaric acid	0.5 ± 0.1	164
Olive pomace	Oleuropein	17.1 ± 0.7	540.5
	3-hydroxytyrosol	4.1 ± 0.1	154.2
	4-hidroxibenzoic acid	3.6 ± 0.1	138.1
	*p*-coumaric acid	1.9 ± 0.1	164
	Rutin	1.8 ± 0.1	610.5
